# Targeting Angiotensin II Type-1 Receptor (AT_1_R) Inhibits the Harmful Phenotype of *Plasmodium*-Specific CD8^+^ T Cells during Blood-Stage Malaria

**DOI:** 10.3389/fcimb.2017.00042

**Published:** 2017-02-16

**Authors:** João L. Silva-Filho, Celso Caruso-Neves, Ana A. S. Pinheiro

**Affiliations:** ^1^Laboratório de Bioquímica e Sinalização Celular, Instituto de Biofísica Carlos Chagas Filho, Universidade Federal do Rio de JaneiroRio de Janeiro, Brazil; ^2^Instituto Nacional de Ciência e Tecnologia em Biologia e Bioimagem, Conselho Nacional de Desenvolvimento Científico e Tecnológico/MCTRio de Janeiro, Brazil; ^3^Instituto Nacional para Pesquisa Translacional em Saúde e Ambiente na Região Amazônica, Conselho Nacional de Desenvolvimento Científico e Tecnológico/MCTRio de Janeiro, Brazil

**Keywords:** angiotensin, AT_1_ receptor, CD8^+^ T cells, blood-stage, malaria

## Abstract

CD8^+^ T-cell response is critical in the pathogenesis of cerebral malaria during blood-stage. Our group and other have been shown that angiotensin II (Ang II) and its receptor AT_1_ (AT_1_R), a key effector axis of renin-angiotensin system (RAS), have immune regulatory effects on T cells. Previously, we showed that inhibition of AT_1_R signaling protects mice against the lethal disease induced by *Plasmodium berghei* ANKA infection However, most of the Ang II/AT_1_R actions were characterized by using only pharmacological approaches, the effects of which may not always be due to a specific receptor blockade. In addition, the mechanisms of action of the AT_1_R in inducing the pathogenic activity of *Plasmodium*-specific CD8^+^ T cells during blood-stage were not determined. Here, we examined how angiotensin II/AT_1_R axis promotes the harmful response of *Plasmodium*-specific CD8^+^ T-cell during blood-stage by using genetic and pharmacological approaches. We evaluated the response of wild-type (WT) and AT_1_R^−/−^ *Plasmodium*-specific CD8^+^ T cells in mice infected with a transgenic PbA lineage expressing ovalbumin; and in parallel infected mice receiving WT *Plasmodium*-specific CD8^+^ T cells were treated with losartan (AT_1_R antagonist) or captopril (ACE inhibitor). Both, AT_1_R^−/−^ OT-I cells and WT OT-I cells from losartan- or captopril-treated mice showed lower expansion, reduced IL-2 production and IL-2Rα expression, lower activation (lower expression of CD69, CD44 and CD160) and lower exhaustion profiles. AT_1_R^−/−^ OT-I cells also exhibit lower expression of the integrin LFA-1 and the chemokine receptors CCR5 and CXCR3, known to play a key role in the development of cerebral malaria. Moreover, AT_1_R^−/−^ OT-I cells produce lower amounts of IFN-γ and TNF-α and show lower degranulation upon restimulation. In conclusion, our results show the pivotal mechanisms of AT_1_R-induced harmful phenotype of *Plasmodium*-specific CD8^+^ T cells during blood-stage malaria.

## Introduction

Malaria is still a major global public health problem with 207 million cases resulting in more than 400,000 deaths annually (Murray et al., 2012; World Health Organization, 2015). The most severe complication, termed cerebral malaria (CM), a neuropathology induced primarily by *Plasmodium falciparum*, is the main cause of death in human malaria (Murray et al., 2012; World Health Organization, 2015). Current therapeutic strategies of CM are limited to anti-parasitic drugs, typically administered late during infection. These pharmacological interventions lack efficacy in many cases, and the mortality rate of CM, even after treatment, remains at 10–20% (Stockman, 2012; Oluwayemi et al., 2013). Better understanding of the parasitological and immunological events leading to the development of CM could aid in the development of improved therapeutic options to treat the disease.

Multiple cell types, including monocytes, macrophages, natural killer cells and CD8^+^ T cells are sequestered within the brain at the onset of experimental CM (ECM) in mice. ECM is a serious neurological syndrome that has many of the clinical and pathological features of human CM in susceptible strains of mice infected with *Plasmodium berghei* ANKA (PbA) (Amante et al., 2010). In this regard, there is mounting evidence suggesting that CD8^+^ T cells are the main effector cells in the development of ECM (Boubou et al., 1999; Belnoue et al., 2002; Nitcheu et al., 2003; Potter et al., 2006; Rénia et al., 2006; Suidan et al., 2008; Hunt et al., 2010; Claser et al., 2011; Haque et al., 2011; Shaw et al., 2015). Because of ethical limitations, it is difficult to determine whether CD8^+^ T cells are involved in the pathogenesis of human CM. But in a systematic post-mortem study of the brains of Malawian children with CM, few CD8^+^ T cells were observed intravascularly in distended capillaries (Dorovini-Zis et al., 2011), which is not inconsistent with the PbA mouse model where the relatively small numbers of sequestered CD8^+^ T cells are difficult to observe by histology (Belnoue et al., 2002). Thus, the key role of CD8^+^ T cells and other immune cells in human disease has been a topic of heated debate. The cellular mechanisms implicated in the damage to the blood–brain barrier seem to involve the degranulation of Granzyme B, perforin, and proinflammatory cytokines such as interferon-γ (IFN-γ), tumor necrosis factor-α (TNF-α) and lymphotoxin-α (LT-α) (Grau et al., 1991; Engwerda et al., 2002; Nitcheu et al., 2003; Potter et al., 2006; Suidan et al., 2008; Claser et al., 2011; Haque et al., 2011). However, evidence of the induction of CD8^+^ T cells specific to blood-stage *Plasmodium* antigens was described only recently (Lau et al., 2011; Howland et al., 2013). Because MHC I-restricted epitopes of *Plasmodium* antigens during blood-stage malaria were not known, transgenic lineages of parasites expressing model epitopes, for which T-cell receptor (TCR) transgenic mice are available, were generated to study the immune response of antigen-specific CD8^+^ T cells (Lundie et al., 2008; Miyakoda et al., 2008). These studies revealed that antigens of blood-stage *P. berghei* parasites are captured and cross-presented by CD8α^+^ dendritic cells to induce activation, proliferation, and effector function of parasite-specific CD8^+^ T cells (Miyakoda et al., 2008; Lundie et al., 2008). In addition, they confirmed that parasite-specific cells are sequestered in the brain and are pathogenic to the host by inducing CM (Lundie et al., 2008; Miyakoda et al., 2008; Howland et al., 2015b). However, the mechanisms that induce the pathogenic activity of parasite-specific CD8^+^ T cells during the blood-stage of *Plasmodium* infection remain poorly understood.

Angiotensin II (Ang II) is a renin–angiotensin system (RAS) effector molecule, which exerts its actions via AT_1_ receptors (AT_1_R) and AT_2_ receptors (AT_2_R), which have been reported to mediate contrasting functions (Basso and Terragno, 2001). Initially, it was thought that the main physiological role of Ang II was to control blood pressure through the regulation of vascular tonus and electrolytic balance (Basso and Terragno, 2001). However, studies have shifted the attention toward its nonclassic effects, and Ang II has been proposed to be central in the inflammatory aspects of different diseases (Bush et al., 2000; Donadelli et al., 2000). Previously, our group and others have demonstrated that T cells express a functional RAS that produces and responds to Ang II mainly via AT_1_R (Kunertradek et al., 1994; Nataraj et al., 1999; Inoue et al., 2006; Guzik et al., 2007; Jurewicz et al., 2007; Hoch et al., 2008; Platten et al., 2009; Silva-Filho et al., 2011, 2013, 2015, 2016; Zhang et al., 2012). AT_1_R expression is upregulated in polyclonal T cells during the blood-stage of PbA infection, and it stimulates the production of perforin and migration/sequestration of polyclonal CD8^+^ T cells in the brain. In turn, CD8^+^ T cells promote cerebral edema, cognitive impairment, and lethal disease (Silva-Filho et al., 2011, 2013). In contrast, more recently, we showed that AT_1_R signaling induces expansion but dampens the activation and exhaustion of antigen-specific CD8^+^ T cells during the effector response to whole-parasite immunization (Silva-Filho et al., 2016). Also, effector cells lacking AT_1_R generate a higher number of memory cells, which is an important factor to limit parasite development in the liver (Silva-Filho et al., 2016). So, it seems that the intrinsic role and function of AT_1_R in CD8^+^ T cells could be stage dependent, decreasing the protective response during immunization with liver-stage parasites but stimulating the harmful response during infection with blood-stage parasites. Indeed, inhibition of AT_1_R only in *Plasmodium*-specific CD8^+^ T cells promoted better control of blood parasitemia and improved survival of mice against the lethal disease induced by blood-stage malaria (Silva-Filho et al., 2016). In agreement, pharmacological blockade of AT_1_R protected mice against ECM and increased survival (Silva-Filho et al., 2013; Gallego-Delgado et al., 2016). Nonetheless, this study characterized the effects of AT_1_R during PbA infection by using only pharmacological approaches, the effects of which may not always be due to a specific receptor blockade. Thus, there is no clear evidence whether the intrinsic role of AT_1_R in CD8^+^ T cells differs following immunization vs. infection. Addressing this issue is important for better delineation of strategies targeting the AT_1_R to improve the protective memory CD8^+^ T cells or to inhibit harmful pro-inflammatory CD8^+^ T-cell responses.

Here, we examined the mechanisms of action of AT_1_R specifically in the *Plasmodium*-specific CD8^+^ T-cell response during blood-stage malaria, by using wild-type (WT; AT_1_R^+/+^) or AT_1_R^−/−^ CD8^+^ T cells from ovalbumin (OVA)-specific TCR transgenic mice (OT-I), which allow the tracking of the antigen-specific CD8^+^ T cell response (Lundie et al., 2008; Miyakoda et al., 2008; Cockburn et al., 2010; Chen and Zavala, 2013; Silva-Filho et al., 2016). In parallel, to complement the genetic approach, we also tested the effects of two drugs that block the AT_1_R response, losartan (AT_1_R antagonist) or captopril (inhibitor of Ang II production), in mice that received WT OT-I cells. For the infection, we used transgenic PbA expressing the C-terminal fragment of OVA (amino acids 150–386) fused to the N-terminus of PbA heat shock protein 70 (Miyakoda et al., 2008). This region in the OVA comprehends the epitope SIINFEKL, which allows the response of the SIINFEKL-specific CD8^+^ T cell population (OT-I cells) to be monitored as the population responding to the blood-stage *Plasmodium* antigen (Miyakoda et al., 2008).

Our results revealed that blockage of AT_1_R by both genetic and pharmacological tools impaired priming of *Plasmodium*-specific CD8^+^ T cells, as observed by reduced expression of activation markers such as CD69, CD160, and CD44. AT_1_R promotes upregulation of interleukin-2 receptor α chain (IL-2Rα) and interleukin-2 (IL-2) production, important for the clonal expansion of antigen-specific CD8^+^ T cells. Moreover, there is a reduction in the expression of the integrin LFA-1 (CD11a), and the chemokine receptors CCR5 and CXCR3 in AT_1_R^−/−^ OT-I cells. The lack of AT_1_R signaling also reduced exhaustion as observed by lower expression of CTLA-4 and LAG-3. Consequently, AT_1_R inhibition decreased cytokine production and degranulation by *Plasmodium*-specific CD8^+^ T cells. Our results demonstrate that, different from the effector response to whole parasite immunization, Ang II, via AT_1_R, stimulates the *Plasmodium*-specific CD8^+^ T-cell response following infection with blood-stage malaria. This brings new perspectives to the protective effect of inhibition of AT_1_R (Silva-Filho et al., 2013, 2016; Gallego-Delgado et al., 2016) and advances the knowledge of the mechanisms involved in the pathogenic activity of parasite-specific CD8^+^ T cells during blood-stage malaria. Moreover, it adds novelty to the functions attributed to the RAS in malaria pathogenesis.

## Materials and methods

### Mice

Six- to eight-week-old male C57BL/6 mice were used in all experiments. WT C57BL/6 mice (CD45.2^+^) were purchased from NCI (Frederick, MD). OT-1 TCR transgenic mice (CD45.1^+^) expressing the TCR specific for OVA_257−264_/K^b^ (SIINFEKL peptide) were kindly provided by Dr. David Sacks (National Institute of Allergy and Infectious Disease, Bethesda, MD). AT_1_R^−/−^ mice (B6.129P2-Agtr1atm1Unc/J; backcrossed to C57BL/6J for seven generations) were purchased from Jackson Laboratory (Bar Harbor, ME).

All animal procedures followed previously described studies (Overstreet et al., 2011; Silva-Filho et al., 2016). Mice that had previously been backcrossed to the CD45.1 C57BL/6 background for more than 10 generations were used from our colony. C57BL/6-*Agtr1a*^*tm1Unc*^ (CD45.1^+^) were crossed to OT-I transgenic C57BL/6 mice and F1 progeny positive for the TCR transgene were crossed back to C57BL/6-*Agtr1a*^*tm1Unc*^ mice (CD45.1^+^) to obtain TCR transgenic mice homozygous for the *Agtr1a*^*tm1Unc*^ targeted mutation (CD45.1^+^). Mice carrying the transgenic TCR (OT-I) were phenotyped by flow cytometry using fluorochrome-conjugated anti-mouse antibodies against Vα2, CD45.1, and CD8. For genotyping, REDExtract-N-Amp tissue PCR kits (Sigma) were used to extract the genomic DNA from tail clippings following the manufacturer's protocol. Primer information and PCRs conditions were obtained following the manufacturer's protocol. Primer sequences were as follow: oIMR0738 (WT forward), TGA GAA CAC CAA TAT CAC TG; oIMR0739 (common), TTC GTA GAC AGG CTT GAG; oIMR6218 (mutant forward), CCT TCT ATC GCC TTC TTG ACG, yielding PCR products of 520 bp if mutant and 483 bp if WT, or both sizes if heterozygote. PCR was performed with the following cycle settings: 94°C for 3 min; 94°C for 30 s; 55°C for 30 s for annealing; and 72°C for elongation for 1 min (total of 35 cycles). All mice were housed, bred, and maintained in the animal care facility at Johns Hopkins University. The Institutional Animal Care and Use Committee of Johns Hopkins University approved the experiments involving mice.

### Parasites

The transgenic *P. berghei* ANKA with truncated C-terminal fragment of OVA (amino acids 150–386) fused to the N-terminal sequence (amino acids 1–5) of the PbA heat shock protein 70 gene was used (Miyakoda et al., 2008). A cryopreserved sample of transgenic *P. berghei* ANKA-infected erythrocytes was kindly provided by Dr. Katsuyuki Yui, Nagasaki University, Nagasaki, Japan. The sample was thawed and injected intraperitoneally into a naive C57BL/6 mouse. Cells were maintained in mice up to seven passages prior to use.

### Adoptive transfer, infection, and treatments

**Figure 1:**
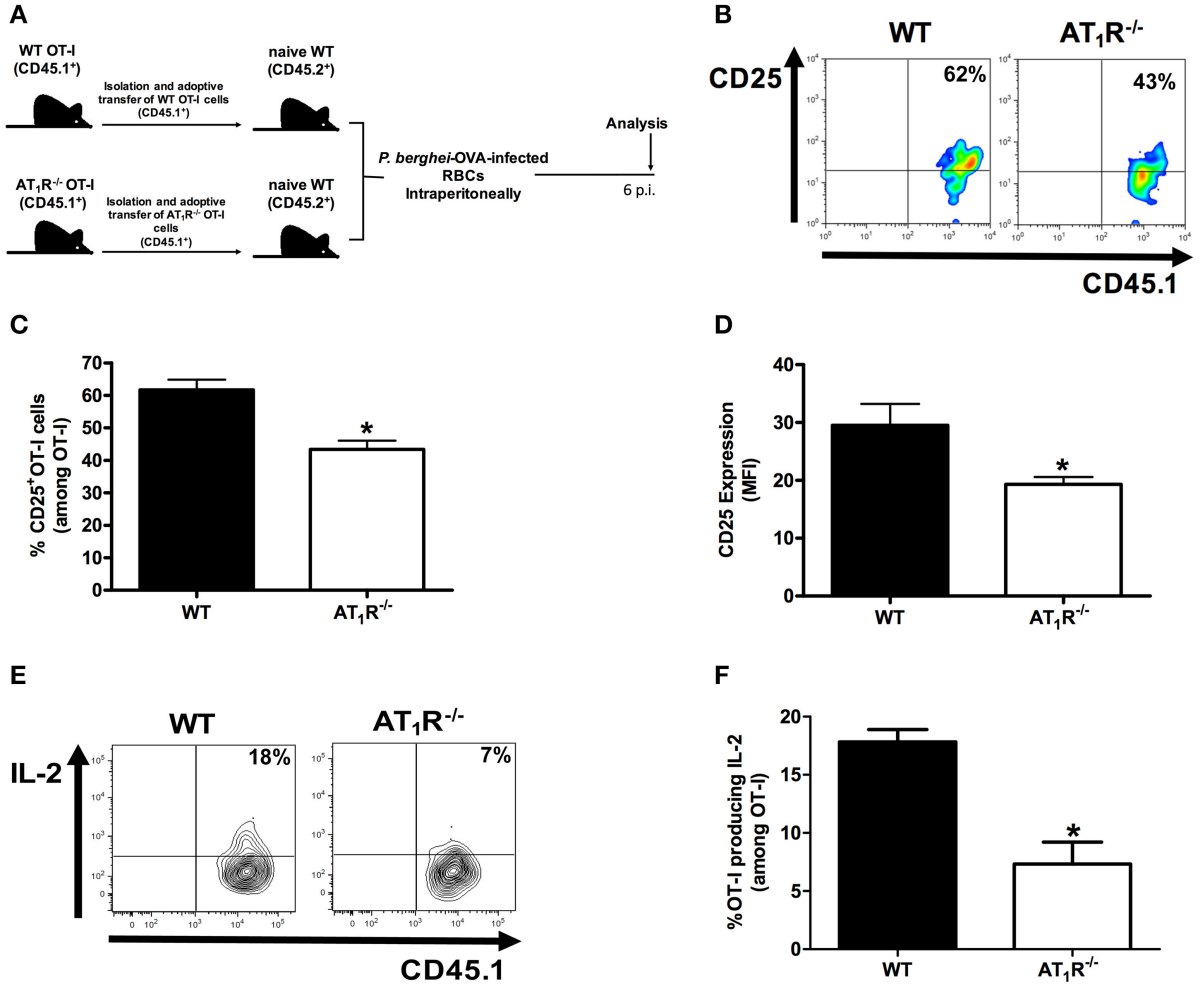
**AT_1_R promotes upregulation of the IL-2Rα chain and IL-2 production in *Plasmodium*-specific CD8^+^ T cells during PbA infection**. AT_1_R^+/+^ or AT_1_R^−/−^ OT-I cells (CD8^+^CD45.1^+^) recovered from the spleen of mice infected with OVA-PbA (CD45.2^+^) were analyzed at day 6 p.i. **(A)** Schematics of the experimental design. 1 × 10^4^ naive WT (AT_1_R^+/+^) or AT_1_R^−/−^ OT-I cells (CD8^+^CD45.1^+^) were adoptively transferred to WT C57Bl/6 mice (CD45.2^+^) recipients 1 day before intraperitoneal injection of 5 × 10^6^ infected red blood cells (RBCs) with *P. berghei* ANKA expressing ovalbumin (OVA-PbA). Mice were euthanized at the indicated time point for recovery and analysis of OT-I cells. **(B,C)** Representative dot plots and percentage of CD25^+^ OT-I cells among total OT-I cells in the spleen on day 6 p.i. (^*^p = 0.0006). **(D)** Expression levels of CD25 in OT-I cells at day 6 p.i. (^*^p = 0.0061). **(E,F)** IL-2 production by WT and AT_1_R^−/−^ OT-I cells was evaluated after 4-h ex vivo re-stimulation with cognate peptide at day 6 p.i. (^*^p = 0.0017). ^*^In relation to WT OT-I cells. Data are means ± SEM of five mice per group and are pooled from three independent experiments with similar results. The gating strategy used for the flow cytometry analysis is indicated in the Materials and Methods section.

**Figure 2:**
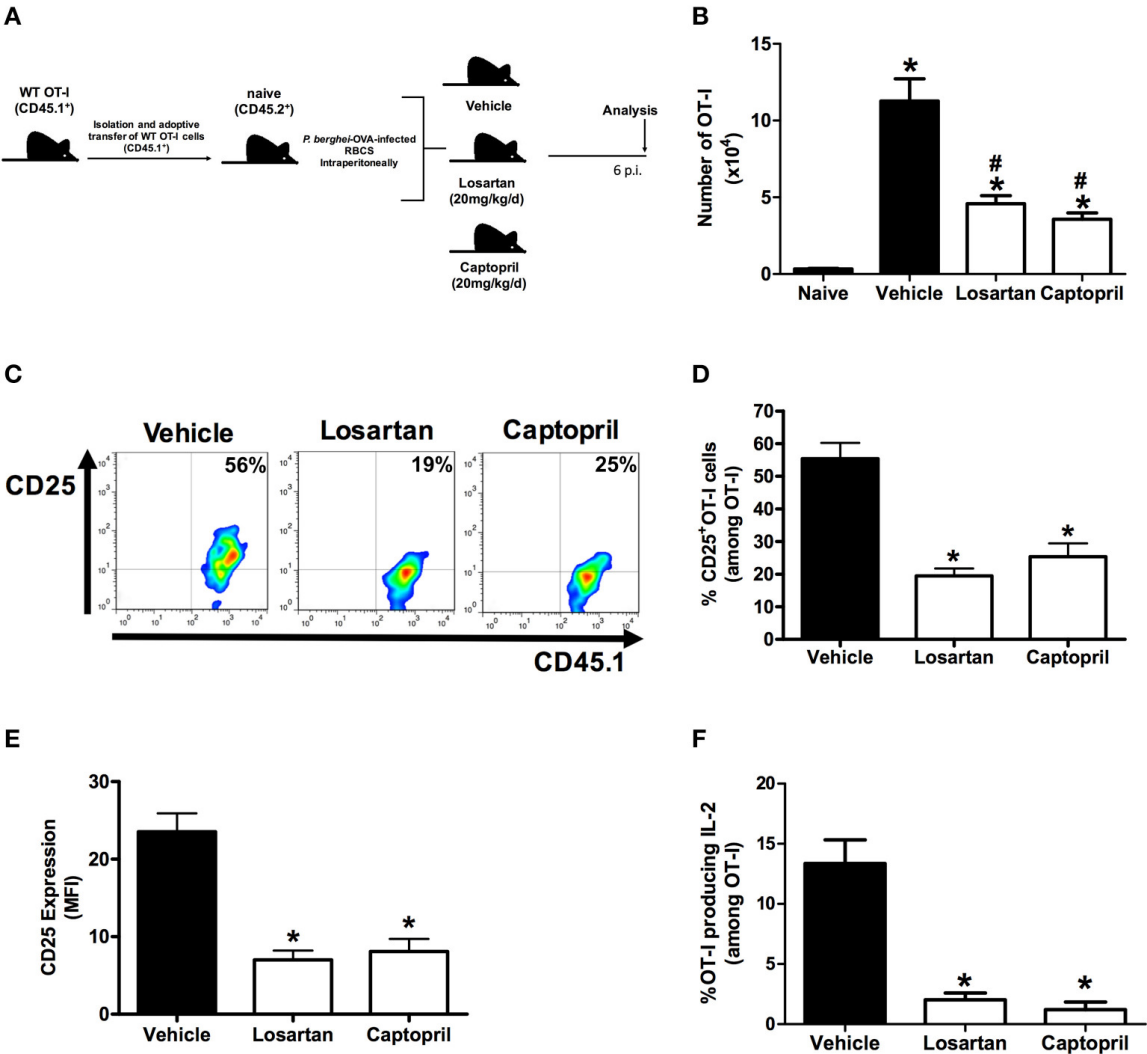
**Losartan and captopril treatments reduce expansion of *Plasmodium*-specific CD8^+^ T cells and IL-2Rα-chain/IL-2 expression**. WT OT-I cells (CD8^+^CD45.1^+^) recovered from the spleen of mice infected with OVA-PbA (CD45.2^+^) treated with vehicle, losartan (20 mg/kg/day) or captopril (20 mg/kg/day) were analyzed at day 6 p.i. **(A)** Schematics of the experimental design. 1 × 10^4^ naive WT (AT_1_R^+/+^) OT-I cells (CD8^+^CD45.1^+^) were adoptively transferred to WT C57Bl/6 mice (CD45.2^+^) recipients 1 day before intraperitoneal injection of 5 × 10^6^ infected red blood cells (RBCs) with *P. berghei* ANKA expressing ovalbumin (OVA-PbA). Mice were separated into three groups: (1) treated with vehicle, (2) treated with 20 mg/kg losartan, an AT_1_ receptor blocker, (3) treated with 20 mg/kg captopril, an angiotensin-converting enzyme inhibitor. Mice were euthanized at the indicated time point for recovery and analysis of OT-I cells. **(B)** Total number of WT OT-I cells per spleen at day 6 post infection in naive, vehicle-, losartan-, and captopril-treated mice infected with OVA-PbA, calculated as the frequencies obtained by CD8^+^CD45.1^+^ staining, multiplied by the total number of cells obtained after spleen excision (^*^p < 0.0001 in relation to naive OT-I cells; #*p* < 0.0001 in relation to WT OT-I cells from infected mice treated with vehicle). Data are means ± SEM of five mice per group and are representative of three independent experiments with similar results. **(C,D)** Representative dot plots and percentage of CD25^+^ OT-I cells among total OT-I cells in the spleen on day 6 post infection (^*^p < 0.0001). **(E)** Expression levels of CD25 in OT-I cells at day 6 post infection (^*^p < 0.0001). **(F)** IL-2 production by WT OT-I cells from the different groups was evaluated after 4-h *ex-vivo* re-stimulation with cognate peptide at day 6 post infection (^*^p < 0.0001). ^*^In relation to WT OT-I cells from infected mice treated with vehicle. Data are means ± SEM of five mice per group and are pooled from three independent experiments with similar results. The gating strategy used for the flow cytometry analysis is indicated in the Materials and Methods section.

For adoptive transfer, cell suspensions from the spleen of WT (AT_1_R^+/+^) and AT_1_R^−/−^ OT-I mice were prepared and pooled. CD8^+^ T cells were purified by negative isolation using magnetic beads according to the manufacturer's instructions (CD8a^+^ T cell isolation kit; Miltenyi Biotech, Paris, France). We obtained >95% CD8^+^CD45.1^+^ T cell (OT-I) enrichments and 10^4^ purified naive WT or AT_1_R^−/−^ OT-I cells (CD45.1^+^) were injected into the tail vein of WT C57BL/6 mice (CD45.2^+^). After 24 h, the recipient mice were infected with OVA-PbA by intraperitoneal injection of 5 × 10^6^ infected red blood cells (Figure 1A). In parallel, mice that received WT OT-I cells were divided into three treatment groups: vehicle, or 20 mg/kg/day losartan, an AT_1_ receptor blocker, or 20 mg/kg/day captopril, an angiotensin-converting enzyme (Figure 2A). Treatments began on the day of infection and were administered by gavage daily for 6 days. The condition of the mice was checked daily, and parasitemia was monitored by microscopic examination of standard blood films. Mice were euthanized at day 6 p.i., at the onset of signs of CM such as hemi- or paraplegia, ataxia, deviation of the head, convulsions, and coma (Martins et al., 2010; Haque et al., 2011; Silva-Filho et al., 2013).

### Lymphocyte isolation

Spleens were collected on day 6 p.i. and single-cell suspensions of lymphocytes were obtained as previously described (Silva-Filho et al., 2016). All lymphocytes were prepared and resuspended in DMEM supplemented with 10% heat-inactivated fetal bovine serum (FBS), 50 mM sodium bicarbonate, 2 mM glutamine, 100 U/ml penicillin, 100 μg/ml streptomycin, and 25 mM HEPES. The numbers of OT-I cells were determined by automated cell counting using the Trypan blue dye exclusion method (viability > 95%) and flow cytometry with anti-CD45.1 and anti-CD8 antibodies.

### *Ex vivo* stimulation and intracellular staining

El4 cells (T cell lymphoma cell line of C57/BL6 [H-2^b^] origin) were pulsed or not with SIINFEKL peptide (10 μg/ml) at 37°C for approximately 1 h. Peptide-coated or control target cells were washed three times and added to lymphocytes harvested from the spleen of mice infected with OVA-PbA. The cells were stimulated for 4 h at 37°C in the presence of 1:400 brefeldin A (GolgiPlug; BD Bioscience) and 1:600 monensin (GolgiStop; BD Bioscience) and anti-CD107a-fluorescein isothiocyanate. Cells were washed twice in cold medium before proceeding to surface and intracellular staining. Intracellular staining was performed using a Cytofix/Cytoperm kit (BD Biosciences) and stained for intracellular cytokines (anti-IFN-γ-PE-Cy7, anti-TNF-α-Pacific blue, and anti-IL-2-APC) according to the manufacturer's protocol. Later, cells were washed and analyzed on an LSR II flow cytometer (BD Bioscience).

### Antibodies and flow cytometry

All antibodies were purchased from eBioscience or BD unless stated otherwise. The following fluorochrome-conjugated monoclonal antibodies were used: CD45.1 (A20); CD8 (53-6.7), CD11a (M17/4), CD25 (PC61.5), CD69 (H1.2F3), CD44 (IM7), CD62L (MEL-14), CD107a (1D4B), CD160 (CNX46-3), CCR5 (HM-CCR5-7A4), CTLA-4 (UC10-4B9), CXCR3 (CXCR3-173), LAG-3 (C9B7W), PD-1 (J43), IFN-γ (XMG1.2), IL-2 (ES6-5H4), TNF-α (MP6-XT22). PerCP/PE/fluorescein isothiocyanate-conjugated IgG1 and IgG2 isotype controls were all purchased from BD Pharmingen. All results were collected with CellQuest software on a FACSCalibur analyzer (Becton Dickinson), and cytokine experiments were acquired on a LSRII flow cytometer (Becton Dickinson). For flow cytometry, cells were incubated with Fc block (anti-mouse CD16 and CD32 antibodies) to block non-specific binding sites for 30 min at 4°C. Later, the cells were washed and incubated with the appropriate concentration of antibodies cited above. IgG isotypes were used as irrelevant antibodies to define positive populations as indicated in the gate strategy (Figure S1). At least 10^5^ cells per sample were acquired. Analysis was performed using FlowJo software (TreeStar). All data were collected and presented in a log scale of fluorescence intensity and presented as plots. The percentage of OT-I lymphocytes was determined in a gate of CD8^+^CD45.1^+^ cells (Figure S1), and each analysis was made in relation to the total OT-I cells (gated on CD8^+^CD45.1^+^ cells), as showed in the gate strategy (Figure S1). The MFI was calculated in the total OT-I cells (gated on CD8^+^CD45.1^+^ cells) considering the fluorescence of the isotype control using the FlowJo software (TreeStar) (Figure S1). Some analyses were also performed in the non-OT-I CD8CD45.2 T cell endogenous population, gated on CD8^+^CD45.1^−^ cells (Figure S1).

### Data analysis

Each experiment was carried out using five animals per group. Data are reported as the means ± SEM of three representative and independent experiments with similar results. For parametric distributions, checked by the Shapiro-Wilk test, differences between the two groups (WT and AT_1_R^−/−^) were compared by a two-tailed Student's *t*-test, or Mann–Whitney test for non-parametric distributions. Differences between three groups (vehicle, losartan, and captopril) were compared by one-way analysis of variance, followed by the Newman–Keuls post-test for parametric distributions, or by Kruskal-Wallis test followed by the Dunn's multiple comparison test for non-parametric distributions. All testes were done using Prism 5 software (GraphPad Software, version 5). The level of significance was set at α = 0.05.

## Results

### AT_1_R promotes expansion of *Plasmodium*-specific CD8^+^ T cells during blood-stage malaria

Previously, we demonstrated that Ang II, via AT_1_R, acts as a co-stimulatory signal for T-cell activation, promotes effector function, adhesion, and migration of T cells to the brain during blood-stage PbA infection (Silva-Filho et al., 2013). In addition, AT_1_R in antigen-specific CD8^+^ T cells regulates expansion, differentiation, and function during effector and memory phases of the response against immunization with radiation-attenuated *Plasmodium* sporozoites (Silva-Filho et al., 2016). Here, to determine if the absence of this receptor results in a difference in the expansion of *Plasmodium*-specific CD8^+^ T cells during infection with blood-stage *Plasmodium* parasite, we evaluated the production of IL-2 and expression of the IL-2Rα-chain (CD25). For this, we crossed transgenic OT-I mice (CD45.1^+^) with mice that lack AT_1_R until the generation of homozygous OT-I mice knockout in the *Agtr1a* gene (AT_1_R^−/−^ OT-I mice CD45.1^+^). The specific response was tracked by adoptively transferring naive WT (AT1R^+/+^) or AT_1_R^−/−^ OT-I cells into WT recipient mice (CD45.2^+^) (Figure 1A). The recipient mice were infected 24 h later with 5 × 10^6^ red blood cells infected with OVA-PbA (Figure 1A). In parallel, mice that received WT OT-I cells were treated with vehicle or 20 mg/kg losartan, an AT_1_ receptor antagonist, or 20 mg/kg captopril, an angiotensin-converting enzyme inhibitor (Figure 2A).

Similar to our previous observations (Silva-Filho et al., 2016), both losartan and captopril treatments inhibited the expansion of OT-I cells (Figure 2B). In non-infected mice (naive), the non-activated OT-1 cells do not proliferate and disappear over time (Figure 2B), confirming that stimulation occurs in an antigen-specific manner (Lundie et al., 2008; Miyakoda et al., 2008; Silva-Filho et al., 2016). Six days after infection, when signs of CM became evident (Martins et al., 2010), the percentage of CD25^+^ cells and expression of CD25, determined by the median fluorescence intensity (MFI), were 30 and 35% lower in the AT_1_R^−/−^ OT-I cells, respectively (Figures 1B–D). In addition, there was a 60% reduction in the amount of IL-2 produced by re-stimulated AT_1_R^−/−^ OT-I cells in comparison with WT OT-I cells (Figures 1E,F). Similar to AT_1_R^−/−^ OT-I cells, WT OT-I cells harvested from infected mice treated with losartan or captopril showed lower CD25 expression and IL-2 production under re-stimulation (Figures 2C–F). These results confirm that AT_1_R stimulates expansion of *Plasmodium*-specific CD8^+^ T cells during blood-stage malaria as well as during radiation-attenuated sporozoite immunization (Silva-Filho et al., 2016), and this effect correlates with the upregulation of IL-2Rα expression and IL-2 production.

### AT_1_R is involved in the exacerbated activation of *Plasmodium*-specific CD8^+^ T cells during blood-stage malaria

Next, we evaluated whether genetic and pharmacological inhibition of AT_1_R affect the exacerbated activation of the effector *Plasmodium*-specific CD8^+^ T cells induced by infection with blood-stage *Plasmodium* parasite. Expression of the activation and effector function markers CD69, CD160, CD44, and CD62L were analyzed at day 6 post infection (p.i.).

**Figure 3:**
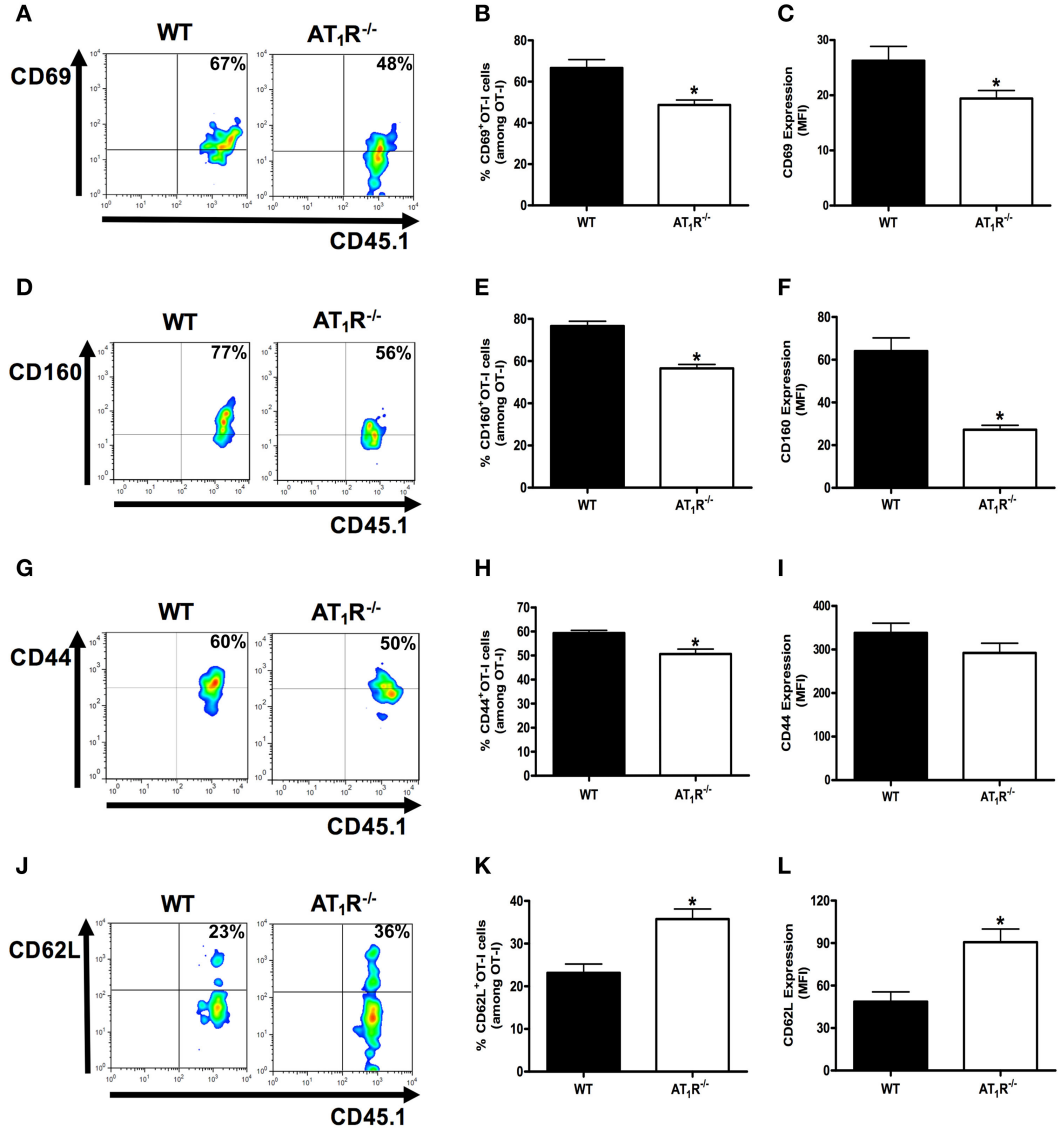
**AT_1_R is a co-stimulatory receptor for activation of *Plasmodium*-specific CD8^+^ T cells**. Percentage of cells expressing the markers of activation CD69, CD160, CD44, and CD62L were evaluated in WT and AT_1_R^−/−^ OT-I cells (CD8^+^CD45.1^+^) recovered from the spleen of mice (CD45.2^+^) infected with OVA-PbA at day 6 p.i. The gating strategy used for the flow cytometry analysis is indicated in the Materials and Methods section. **(A,B)** Representative dot plots and percentage of CD69^+^ OT-I cells among total OT-I cells in the spleen (^*^p = 0.0023). **(C)** Expression of CD69 was evaluated, based on MFI analysis, in WT and AT_1_R^−/−^ OT-I cells (CD8^+^CD45.1^+^) recovered from the spleen of infected recipient mice (CD45.2^+^) at day 6 p.i. (^*^p = 0.0306). **(D,E)** Representative dot plots and percentage of CD160^+^ OT-I cells among total OT-I cells in the spleen (^*^p < 0.0001). **(F)** Expression of CD160 was evaluated, based on MFI analysis, in WT and AT_1_R^−/−^ OT-I cells (CD8^+^CD45.1^+^) recovered from the spleen of infected recipient mice (CD45.2^+^) at day 6 p.i. (^*^p < 0.0001). ^*^In relation to WT OT-I cells. Data are means ± SEM of five mice per group and are pooled from three independent experiments with similar results. **(G,H)** Representative dot plots and percentage of CD44^+^ OT-I cells among total OT-I cells in the spleen (^*^p = 0.0031). **(I)** Expression of CD44 was evaluated, based on MFI analysis, in WT and AT_1_R^−/−^ OT-I cells (CD8^+^CD45.1^+^) (*p* = 0.2405). **(J,K)** Representative dot plots and percentage of CD62L^+^ OT-I cells among total OT-I cells in the spleen (^*^p = 0.0007). **(L)** Expression of CD62L was evaluated, based on MFI analysis, in WT and AT_1_R^−/−^ OT-I cells (CD8^+^CD45.1^+^) (^*^p = 0.0011). ^*^In relation to WT OT-I cells. Data are means ± SEM of five mice per group and are pooled from three independent experiments with similar results.

**Figure 4:**
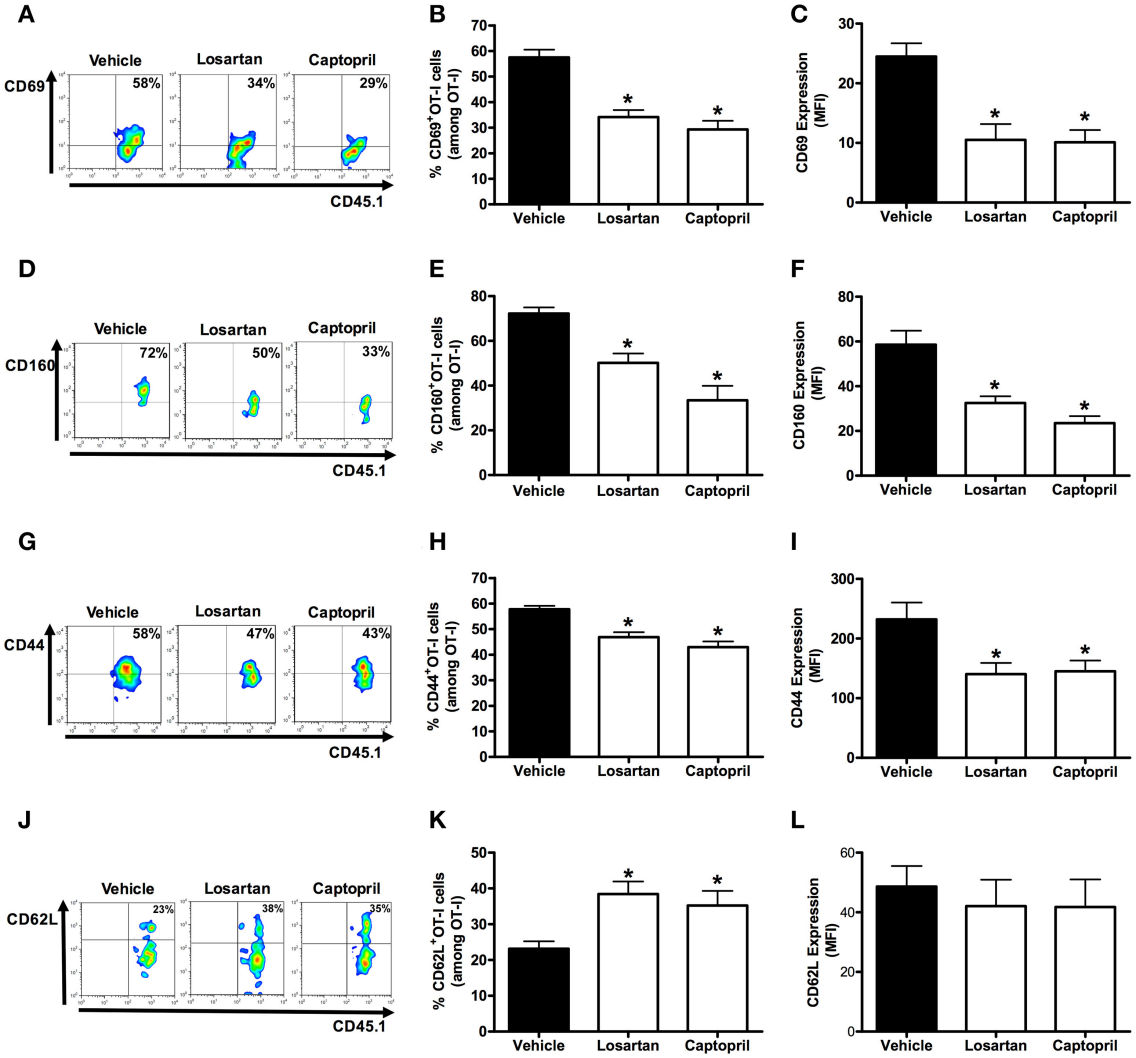
**Losartan and captopril treatments reduce activation of *Plasmodium*-specific CD8^+^ T cells**. Percentage of cells expressing the markers of activation CD69, CD160, CD44, and CD62L were evaluated at day 6 post infection in WT OT-I cells (CD8^+^CD45.1^+^) recovered from the spleen of recipient mice (CD45.2^+^) infected with OVA-PbA and treated with vehicle, losartan (20 mg/kg/day) or captopril (20 mg/kg/day). The gating strategy used for the flow cytometry analysis is indicated in the Materials and Methods section. **(A–C)** Representative dot plots, percentage of CD69^+^ OT-I cells among total OT-I cells in the spleen (^*^p < 0.0001), and expression of CD69 (^*^p = 0.0003). **(D–F)** Representative dot plots, percentage of CD160^+^ OT-I cells among total OT-I cells in the spleen (^*^p < 0.0001), and expression of CD160 (^*^p = 0.0001). **(G–I)** Representative dot plots, percentage of CD44^+^ OT-I cells among total OT-I cells in the spleen (^*^p < 0.0001), and expression of CD44 was evaluated (^*^p = 0.0112). **(J–L)** Representative dot plots, percentage of CD62L^+^ OT-I cells among total OT-I cells in the spleen (^*^p = 0.0047), and expression of CD62L (*p* = 0.7873). ^*^In relation to WT OT-I cells from infected mice treated with vehicle. Data are means ± SEM of five mice per group and are pooled from three independent experiments with similar results.

The percentage and expression of CD69 were 35 and 33% lower in AT_1_R^−/−^ OT-I cells than WT controls, respectively (Figures 3A–C). Losartan and captopril treatments also inhibited CD69 upregulation in WT OT-I cells (Figures 4A–C). Upregulation of CD160 expression was inhibited 58% in AT_1_R^−/−^ OT-I cells compared with the WT OT-I cells (Figures 3D–F). In turn, WT OT-I cells harvested from infected mice that received losartan or captopril treatment showed a similar decrease in CD160 frequency and expression (Figures 4D–F). We also verified a slight decrease in the percentage of CD44^+^ AT_1_R^−/−^ OT-I cells (Figures 3G–I). CD62L expression decreases in the effector population [40]. In this regard, CD62L expression is significantly lower in WT OT-I cells (MFI, 48.6 ± 6.7) than in AT_1_R^−/−^ OT-I cells (MFI, 90.7 ± 8.7) (Figures 3J–L). WT OT-I cells exposed to losartan or captopril treatment showed reduced CD44 expression and a higher percentage of CD62L^+^ cells (Figures 4G–L). The reduced clonal expansion and expression of activation markers in OT-I cells lacking AT_1_R signaling reveal its influence in the priming of *Plasmodium*-specific CD8^+^ T cells during the blood-stage of PbA infection.

To make sure that functional antigen presentation, and then T-cell activation, are not affected by WT or AT_1_R^−/−^ OT-I cells, we analyzed endogenous CD8^+^ T cells, gated on CD8^+^CD45.1^−^ cells (Figure S1). As previously demonstrated (Miyakoda et al., 2008), a lower but significant level of activation was detected in non-OT-I CD8^+^ T cells (CD8^+^CD45.1^−^) from infected mice in relation to cells from an uninfected host (naive) (Figure S2). Importantly, changes in the expression of CD69, CD44, CD160, and CD62L in these cells were similar in mice that received either WT (WT → WT) or AT_1_R^−/−^ OT-I cells (AT_1_R^−/−^ → WT) (Figure S2). These results confirm that WT and AT_1_R^−/−^ OT-I cells respond in similar environments, where the presentation of the parasite antigens is the same.

### AT_1_R induces exhaustion of *Plasmodium*-specific CD8^+^ T cells during the blood-stage malaria

It is known that effector function of CD8^+^ T cells can be reduced by increasing the expression of inhibitory receptors, which are also referred to as markers of cellular exhaustion (Baitsch et al., 2012; Legat et al., 2013). They are upregulated during differentiation to effector cells and function as a negative feedback mechanism. Thus, they are also used for monitoring the status of activation and differentiation of effector CD8^+^ T cells. Here, to better understand the influence of AT_1_R in the activation and differentiation of effector antigen-specific CD8^+^ T cells, we verified the expression of the exhaustion markers CTLA-4, PD1, and LAG-3 (Scheipers and Reiser, 1998; Zha et al., 2004; Richter et al., 2009).

**Figure 5:**
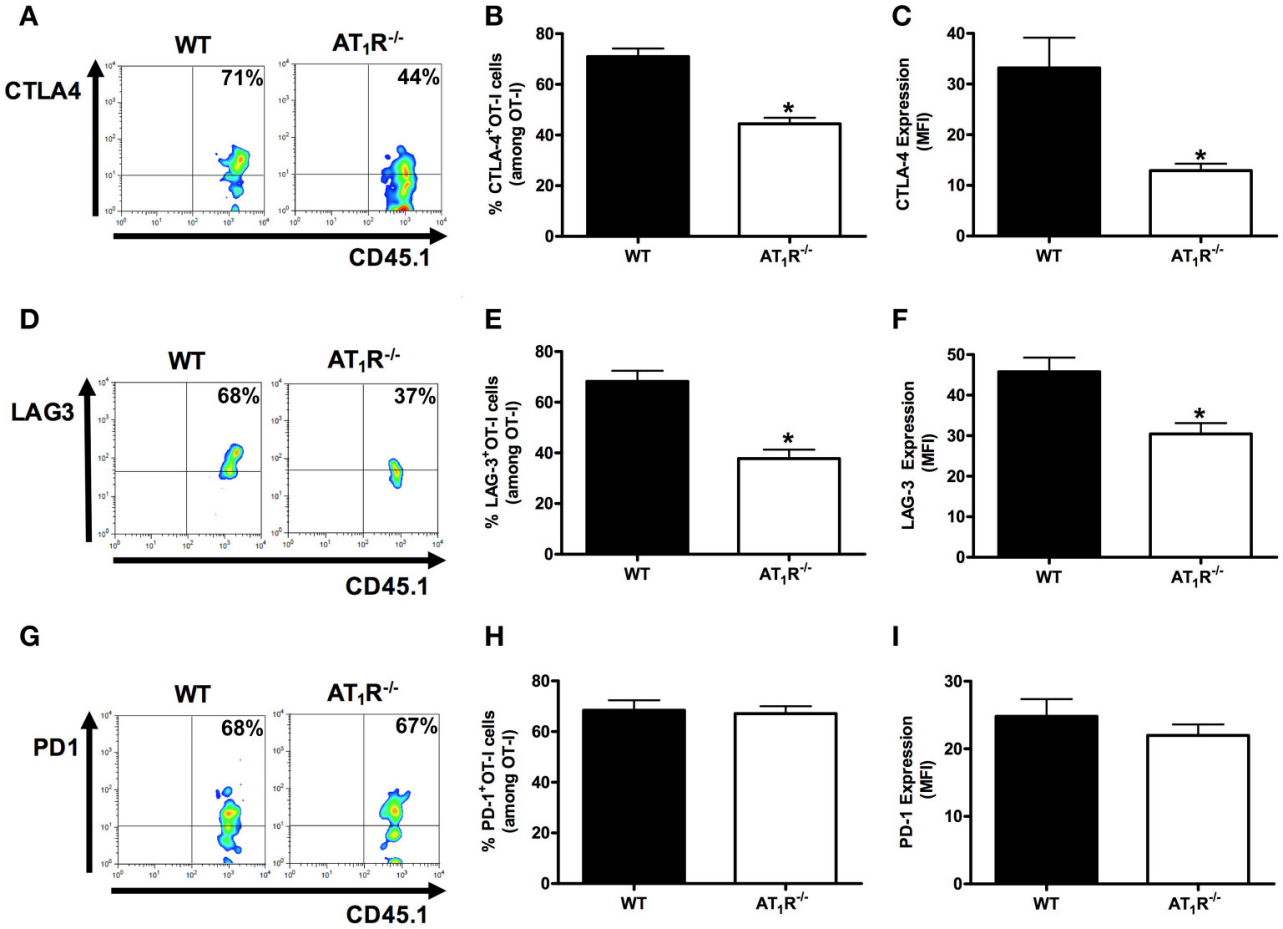
**AT_1_R induces exhaustion of *Plasmodium*-specific CD8^+^ T cells**. The percentage of cells expressing exhaustion markers, PD-1, LAG-3, and CTLA-4, were evaluated in WT and AT_1_R^−/−^ OT-I cells (CD8^+^CD45.1^+^) isolated from the spleen of mice (CD45.2^+^) infected with OVA-PbA at day 6 p.i. The gating strategy used for the flow cytometry analysis is indicated in the Materials and Methods. **(A–C)** Representative dot plots, percentage of CTLA-4^+^ OT-I cells among total OT-I cells in the spleen (^*^p < 0.0001), and expression of CTLA-4 (^*^p < 0.0001). **(D–F)** Representative dot plots, percentage of LAG-3^+^ OT-I cells among total OT-I cells in the spleen (^*^p < 0.0001) and expression of LAG-3 (^*^p = 0.0018). **(G–I)** Representative dot plots, percentage of PD-1^+^ OT-I cells among total OT-I cells in the spleen (*p* = 0.7826), and expression of PD-1 (*p* = 0.3596). ^*^In relation to WT OT-I cells. Data are means ± SEM of five mice per group and are representative of three independent experiments with similar results for each indicated time point.

**Figure 6:**
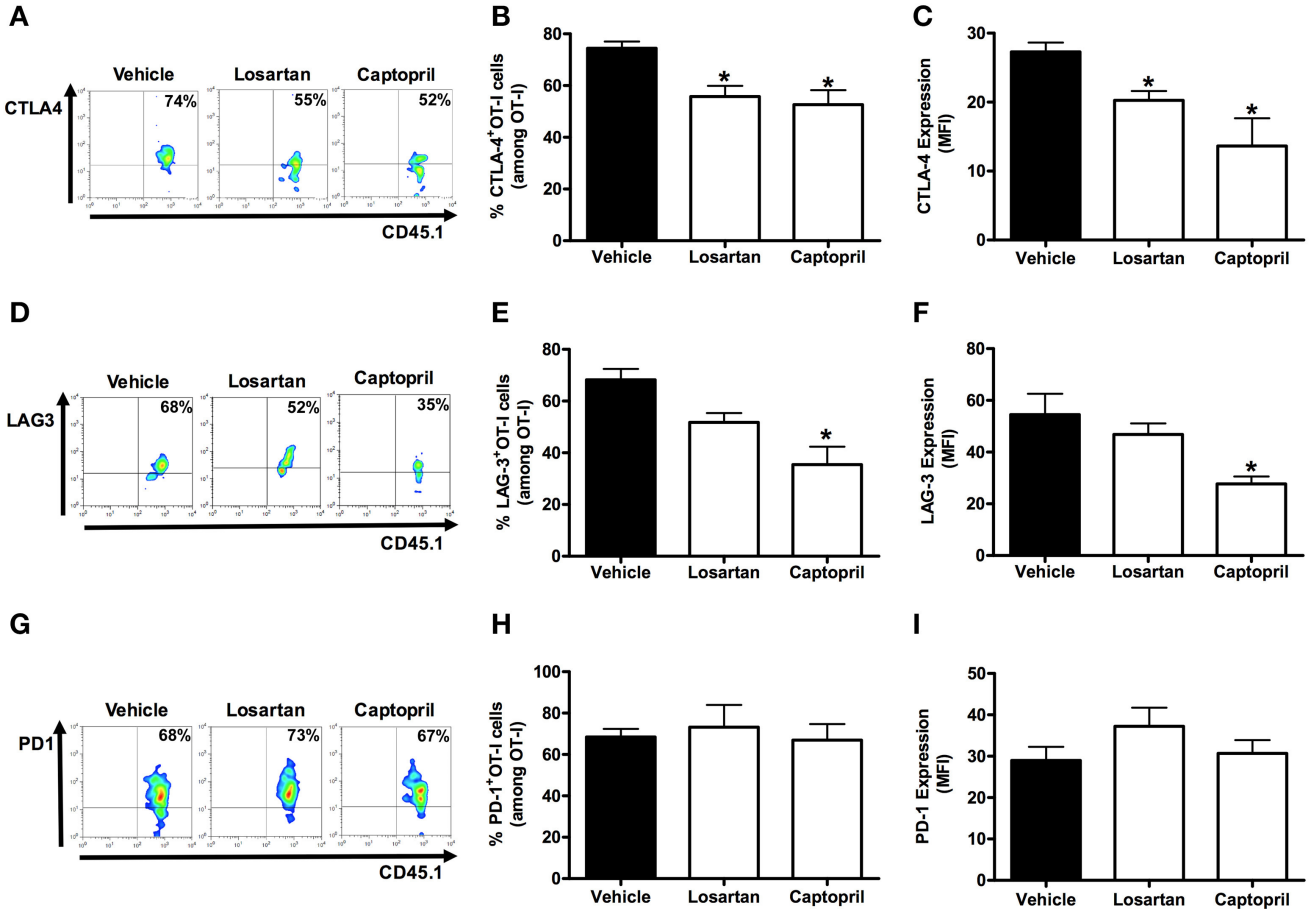
**Losartan and captopril treatments reduce exhaustion of *Plasmodium*-specific CD8^+^ T cells**. Percentage of cells expressing exhaustion markers, PD-1, LAG-3, and CTLA-4, were evaluated in WT OT-I cells (CD8^+^CD45.1^+^) isolated from the spleen of recipient mice (CD45.2^+^) infected with OVA-PbA and treated with vehicle, losartan (20 mg/kg/day), or captopril (20 mg/kg/day). The gating strategy used for the flow cytometry analysis is indicated in the Materials and Methods section. **(A–C)** Representative dot plots, percentage of CTLA-4^+^ OT-I cells among total OT-I cells in the spleen (^*^p = 0.0012), and expression of CTLA-4 (^*^p = 0.0008). **(D–F)** Representative dot plots, percentage of LAG-3^+^ OT-I cells among total OT-I cells in the spleen (^*^p = 0.001), and expression of LAG-3 (^*^p = 0.0137). **(G–I)** Representative dot plots, percentage of PD-1^+^ OT-I cells among total OT-I cells in the spleen (*p* = 0.8294), and expression of PD-1 (*p* = 0.3413). ^*^In relation to WT OT-I cells from infected mice treated with vehicle. Data are means ± SEM of five mice per group and are representative of three independent experiments with similar results for each indicated time point.

Figures 5, 6 shows that, at day 6 p.i., the percentage and expression of CTLA-4 and LAG-3 were lower in AT_1_R^−/−^ OT-I cells and WT OT-I cells exposed to captopril treatment. In contrast, in WT OT-I cells from mice infected treated with losartan, only the frequency and expression of CTLA-4 were reduced (Figure 6). PD-1 expression and frequency were not changed in any OT-I cells lacking AT_1_R signaling (Figures 5, 6). Thus, AT_1_R signaling in *Plasmodium*-specific CD8^+^ T cells promotes exhaustion of the responding population, along with higher activation.

### AT_1_R upregulates integrin and chemokine receptors in *Plasmodium*-specific CD8^+^ T cells

Previously, we showed the key role of Ang II/AT_1_R axis in the upregulation of CD11a, and the chemokine receptors CCR2 and CCR5 in polyclonal T cells during the blood stage of PbA infection (Silva-Filho et al., 2013). Here, we tested whether AT_1_R expressed in *Plasmodium*-specific CD8^+^ T cells modulates the expression of molecules involved in the migration and sequestration of pathogenic CD8^+^ T cells in inflamed tissues during severe malaria, such as LFA-1 (CD11a), CCR5, and CXCR3 (Falanga and Butcher, 1991; Belnoue, 2003; Sarfo et al., 2004; Hansen et al., 2007; Nie et al., 2009).

**Figure 7:**
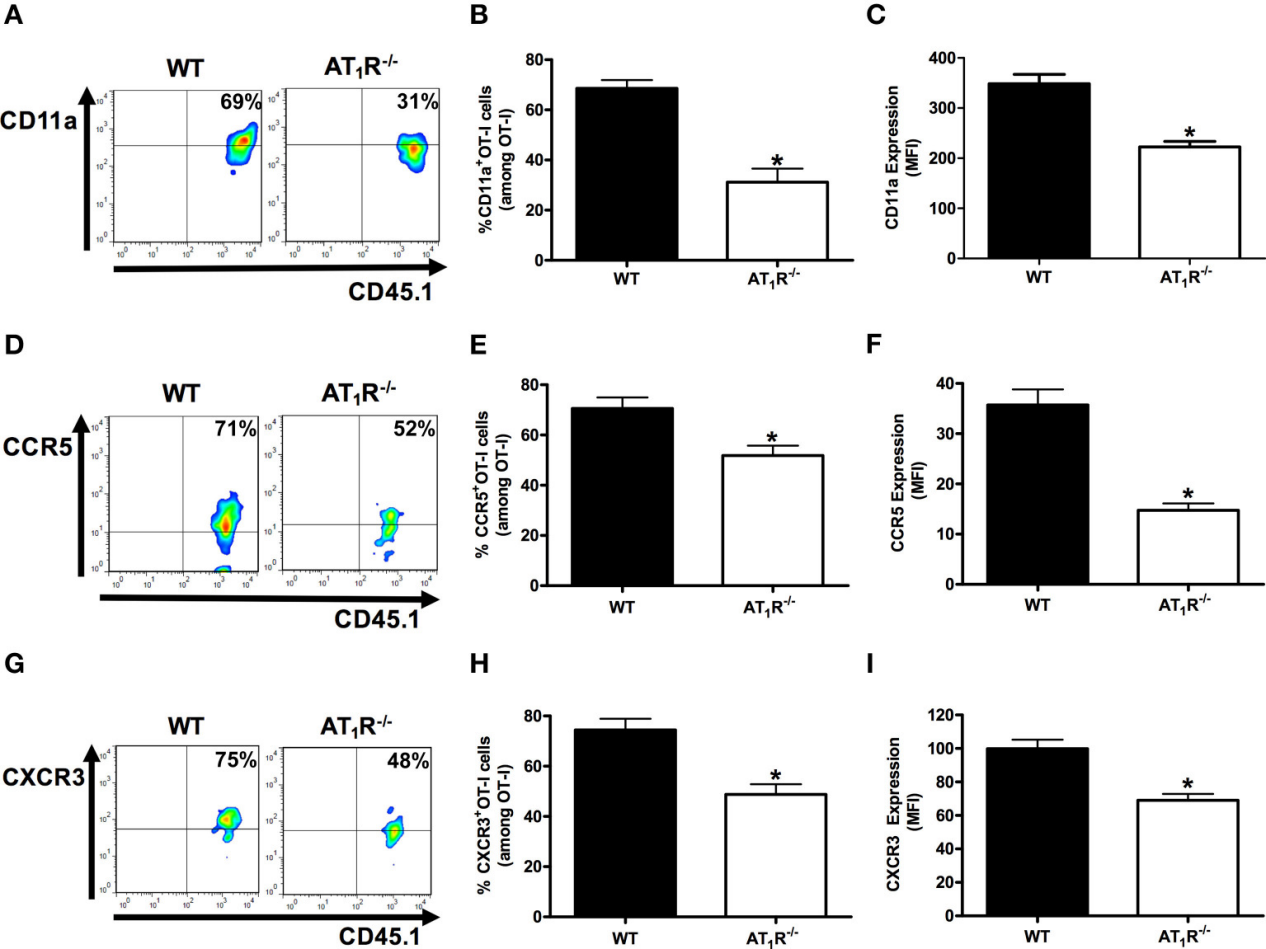
**AT_1_R upregulates CD11a and chemokine receptors in *Plasmodium*-specific CD8^+^ T cells during blood-stage PbA infection**. The percentage of cells expressing CD11a, CCR5, and CXCR3 was evaluated in WT and AT_1_R^−/−^ OT-I cells (CD8^+^CD45.1^+^) harvested from the spleen of recipient mice (CD45.2^+^) infected with OVA-PbA and analyzed at day 6 p.i. The gating strategy used for the flow cytometry analysis is indicated in the Materials and Methods section. **(A–C)** Representative dot plots, percentage of CD11a^+^ OT-I cells among total OT-I cells in the spleen (^*^p < 0.0001), and expression of CD11a (^*^p < 0.0001). **(D–F)** Representative dot plots, percentage of CCR5^+^ OT-I cells among total OT-I cells in the spleen (^*^p = 0.0041) and expression of CCR5 (^*^p < 0.0001). **(G–I)** Representative dot plots, percentage of CXCR3^+^ OT-I cells among total OT-I cells in the spleen (^*^p = 0.0004), and expression of CXCR3 (^*^p = 0.0001). ^*^In relation to WT OT-I cells. Data are means ± SEM of four mice per group and are representative of three independent experiments with similar results.

**Figure 8:**
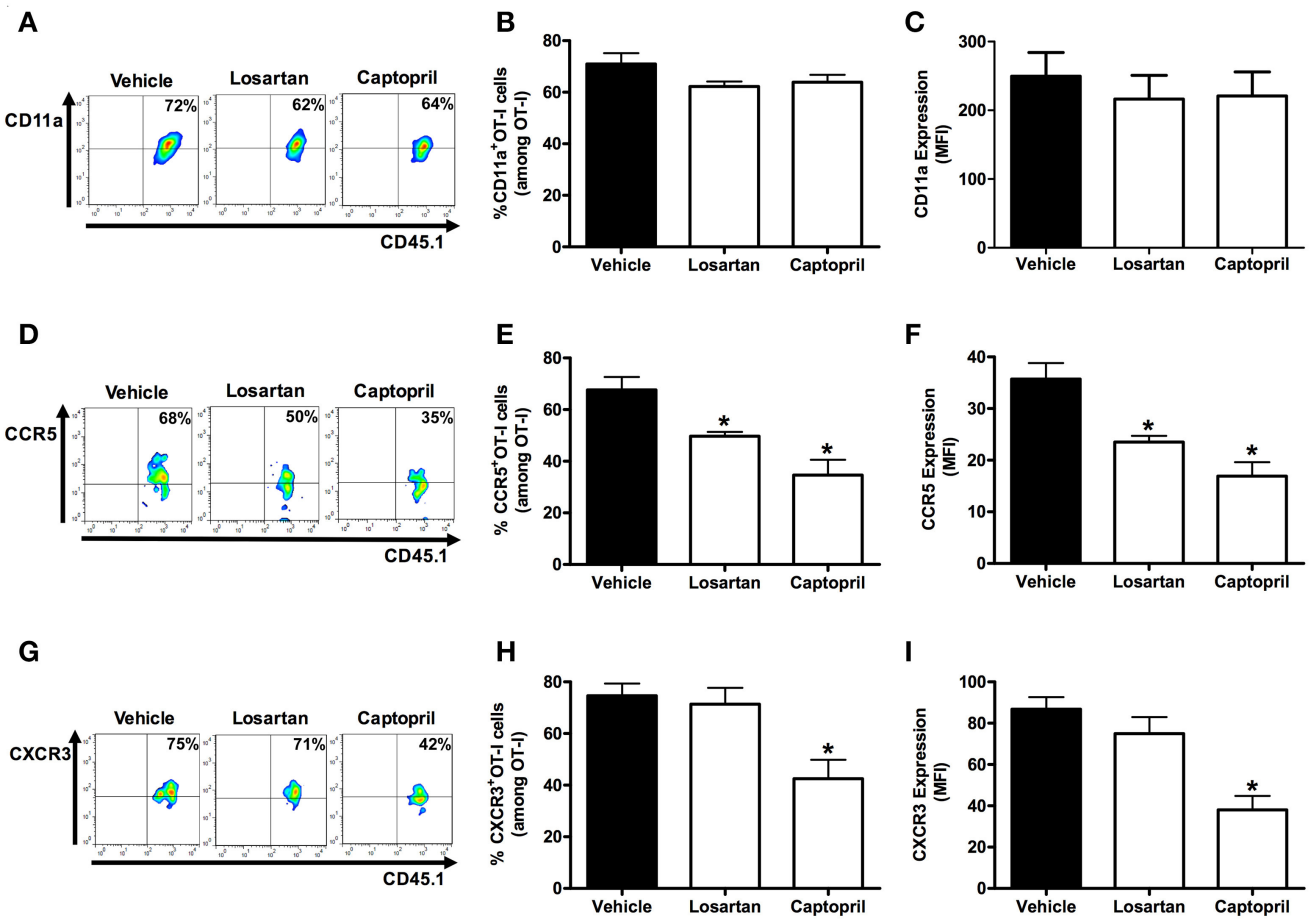
**Losartan and captopril treatments reduce chemokine receptor expression in *Plasmodium*-specific CD8^+^ T cells**. Percentage of cells expressing CD11a, CCR5, and CXCR3 were evaluated in WT OT-I cells (CD8^+^CD45.1^+^) harvested from the spleen of recipient mice (CD45.2^+^) infected with OVA-PbA and treated with vehicle, losartan (20 mg/kg/day), or captopril (20 mg/kg/day). The gating strategy used for the flow cytometry analysis is indicated in the Materials and Methods section. **(A–C)** Representative dot plots, percentage of CD11a^+^ OT-I cells among total OT-I cells in the spleen (*p* = 0.1721), and expression of CD11a (*p* = 0.7627). **(D–F)** Representative dot plots, percentage of CCR5^+^ OT-I cells among total OT-I cells in the spleen (^*^p = 0.0005), and expression of CCR5 (^*^p = 0.0001). **(G–I)** Representative dot plots, percentage of CXCR3^+^ OT-I cells among total OT-I cells in the spleen (^*^p = 0.0053), and expression of CXCR3 (^*^p = 0.0001). ^*^In relation to WT OT-I cells from infected mice treated with vehicle. Data are means ± SEM of four mice per group and are representative of three independent experiments with similar results.

Figure 7 shows that AT_1_R^−/−^ OT-I cells from mice infected with OVA-PbA significantly express lower amounts of CD11a (55% less), CCR5 (27% less), and CXCR3 (36% less) in relation to WT controls. Losartan treatment inhibited only the upregulation of CCR5 expression, whereas captopril inhibited the increase of CCR5 and CXCR3 expression in WT OT-I cells (Figure 8). These results imply that AT_1_R expressed in *Plasmodium*-specific CD8^+^ T cells promotes the upregulation of CD11a and chemokine receptors involved in the accumulation of these cells in inflamed tissues during blood-stage malaria, which agrees with the pathogenic role of AT_1_R during cerebral malaria (Silva-Filho et al., 2016).

### AT_1_R stimulates cytokine production and degranulation by *Plasmodium*-specific CD8^+^ T cells during the blood-stage malaria

So far, AT_1_ receptor is important for a higher activation, exhaustion, and expression of adhesion molecules/chemokine receptors in *Plasmodium*-specific CD8^+^ T cells. Previously, we showed that losartan and captopril impaired IFN-γ, IL-17 by CD4^+^ T cells, and perforin production by CD8^+^ T cells during PbA infection (Silva-Filho et al., 2013). In addition, effector antigen-specific CD8^+^ T cells lacking AT_1_R show lower polyfunctional capacity (Silva-Filho et al., 2016). Then, we evaluated the specific influence of AT_1_R in the production of cytokines and degranulation by *Plasmodium*-specific CD8^+^ T cells after *ex-vivo* stimulation with SIINFEKL-pulsed EL4 target cells.

**Figure 9:**
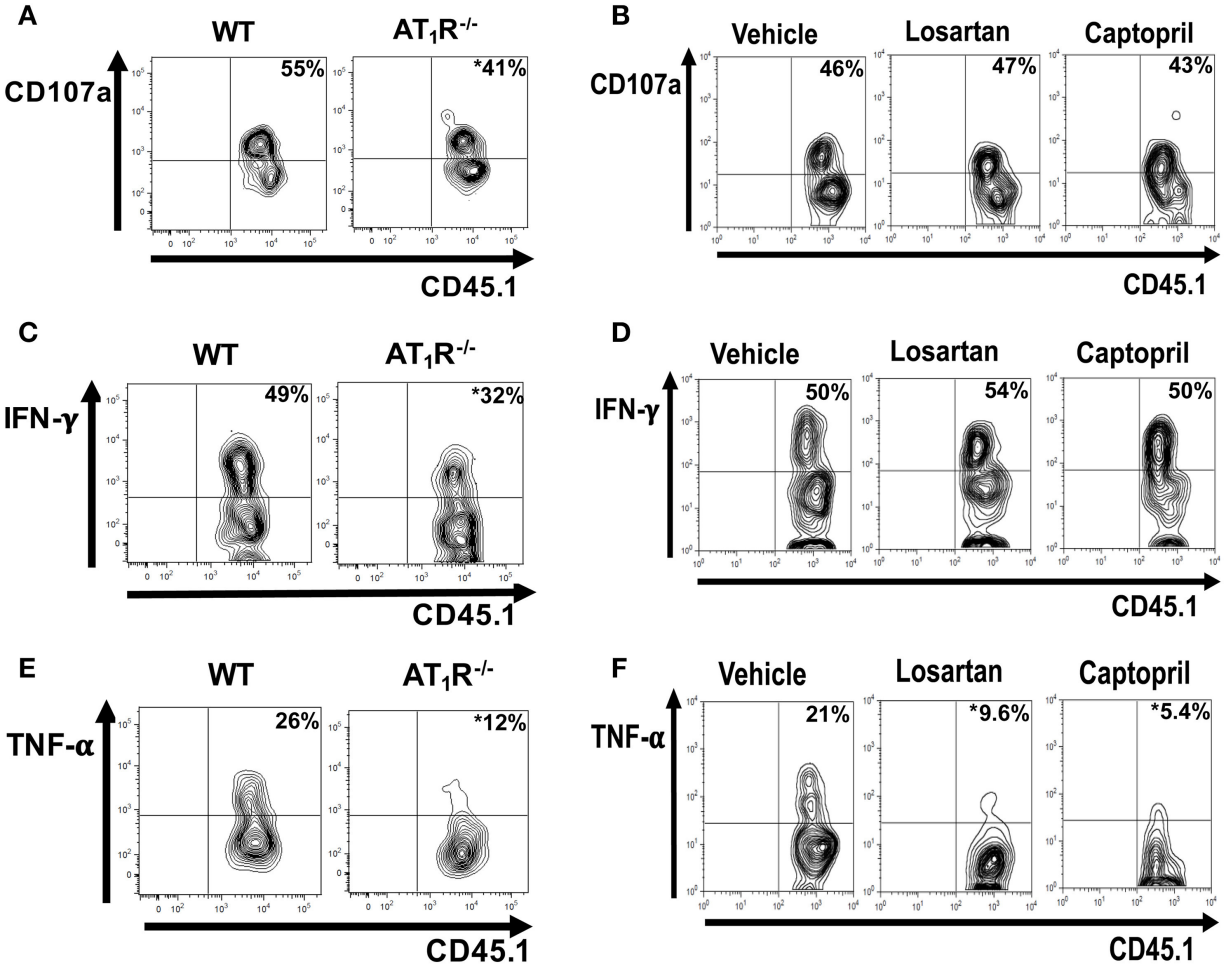
**AT_1_R promotes cytokine production and degranulation of effector *Plasmodium*-specific CD8^+^ T cells**. 1 × 10^4^ naive WT or AT_1_R^−/−^ CD45.1^+^ OT-I cells were adoptively transferred to WT C57BL/6 (CD45.2^+^) recipients 1 day before infection with 5 × 10^6^ RBCs infected with OVA-PbA. WT OT-I cells (CD8^+^CD45.1^+^) were also recovered from the spleen of mice infected with OVA-PbA (CD45.2^+^) treated with vehicle, losartan (20 mg/kg/day) or captopril (20 mg/kg/day). Six days post infection, cytokine production was evaluated after 4-h *ex-vivo* re-stimulation with SIINFEKL peptide-coated target cells. **(A,B)** Representative dot plots of CD107a^+^ OT-I cells among total OT-I cells in the spleen. **(C,D)** Representative dot plots of IFN-γ^+^ OT-I cells among total OT-I cells in the spleen. **(E,F)** Representative dot plots of TNF-α^+^ OT-I cells among total OT-I cells in the spleen. ^*^p < 0.05 in relation to WT OT-I cells or WT OT-I cells from mice treated with vehicle. Data are means ± SEM of four mice per group and are representative of three independent experiments with similar results.

Analysis of CD107a, a lysosomal-associated membrane protein associated with cytolytic granules that can be detected on the surface of T cells following degranulation, showed that degranulation was impaired in AT_1_R^−/−^ OT-I cells (Figure 9A). Moreover, AT_1_R^−/−^ OT-I cells produce lower amounts of IFN-γ and TNF-α (Figures 9C,E). Treatment of infected mice with losartan or captopril only decreased the production of TNF-α by the WT OT-I cells (Figure 9). Together, our results show that Ang II, via AT_1_R, promotes a harmful phenotype of *Plasmodium*-specific CD8^+^ T cells during blood-stage malaria by inducing an exacerbated effector response, represented by a higher activation, capacity to migrate to inflamed tissues, cytokine production and degranulation.

## Discussion

Accumulating evidences show that CD8^+^ T cells are the main effector cells in the development of CM in murine models (Boubou et al., 1999; Belnoue et al., 2002; Nitcheu et al., 2003; Potter et al., 2006; Rénia et al., 2006; Suidan et al., 2008; Hunt et al., 2010; Claser et al., 2011; Haque et al., 2011; Shaw et al., 2015). It is known that Ang II binds to two different receptors, AT_1_R and AT_2_R, but we verified that only AT_1_R is upregulated in CD8^+^ T cells and mediates the effects of Ang II during PbA infection (Silva-Filho et al., 2011, 2013, 2016). AT_1_R acts as a co-stimulatory receptor for T-cell activation, promoting perforin expression and sequestration of CD8^+^ T cells in the brain, a cellular mechanism involved in cerebral edema and behavioral impairment during the blood stage of PbA infection, as well as in other models of diseases (Guzik et al., 2007; Platten et al., 2009; Silva-Filho et al., 2011, 2013; Zhang et al., 2012). In this work, using a transgenic parasite lineage expressing the model antigen ovalbumin (OVA-PbA) (Miyakoda et al., 2008), we further evaluated the role of AT_1_R expressed in blood-stage *Plasmodium*-specific CD8^+^ T cells. We demonstrated that Ang II/AT_1_R is a stimulatory axis for activation, exhaustion, migration and effector function of *Plasmodium*-specific CD8^+^ T cells. These observations suggest the role of AT_1_R in stimulating a harmful CD8^+^ T-cell response that accelerates lethal CM (Silva-Filho et al., 2013, 2016; Gallego-Delgado et al., 2016) and expand the knowledge of the RAS in malaria pathogenesis.

Previous studies using transgenic lineages of parasites expressing model epitope in combination with TCR transgenic mice (Lundie et al., 2008; Miyakoda et al., 2008), revealed that antigens of blood-stage *P. berghei* parasites are captured and cross-presented by CD8α^+^ dendritic cells and stimulate antigen-specific CD8^+^ T cells response (Lundie et al., 2008; Miyakoda et al., 2008; Lau et al., 2011; Howland et al., 2013, 2015a). In addition, highly activated parasite-specific CD8^+^ T cells are sequestered in the brain and recognize antigens presented by endothelial cells (Grau et al., 1991; Boubou et al., 1999; Engwerda et al., 2002; Nitcheu et al., 2003; Potter et al., 2006; Suidan et al., 2008; Claser et al., 2011; Haque et al., 2011; Shaw et al., 2015). Recently, it was identified that Vβ8.1^+^ CD8^+^ T cells recognize the H-2D^b^-restricted epitope SQLLNAKYL from glideosome-associated protein 50 from PbA (Howland et al., 2013). These cells are enriched in the brain during ECM (Boubou et al., 1999; Belnoue et al., 2002) and they are able to damage the blood–brain barrier and mediate CM (Howland et al., 2013, 2015a). In human CM, because of ethical limitations, the key role of CD8^+^ T cells in human disease is difficult to determine. However, there is evidence that CD8^+^ T cells play an important role. In a systematic post-mortem study of the brains of Malawian children with CM, few CD8^+^ T cells were observed intravascularly in distended capillaries (Dorovini-Zis et al., 2011), similar to mice infected with PbA mouse, where the relatively small numbers of sequestered CD8^+^ T cells are difficult to observe by histology (Belnoue et al., 2002). CXCL10, whose primary function is to mediate the migration of activated CD8^+^ T cells, has been associated with CM. CXCL10 was the only biomarker quantified in post-mortem serum, that was statistically different between Ghanaian children who had died of CM vs. malarial anemia (Armah et al., 2007). In Indian patients, high levels of CXCL10 were also associated with a higher risk of CM mortality (Jain et al., 2008; Wilson et al., 2011). In addition, a single nucleotide polymorphism in the CXCL10 gene promoter was associated with increased gene expression and a higher risk of CM (Wilson et al., 2013). The discovery of blood-stage *P. falciparum* MHC class I epitopes would clarify whether pathogenic CD8^+^ T cells are induced and important during human CM.

Here, using known pharmacological tools we confirmed that AT_1_R is important for the clonal expansion of the effector *Plasmodium*-specific CD8^+^ T cells during blood-stage malaria. It has been demonstrated that IL-2 signaling is important for CD8^+^ T-cell proliferation as shown in different models (Nataraj et al., 1999; Inoue et al., 2006; D'Cruz et al., 2009). Thus, as a correlated mechanism, we verified that AT_1_R increases IL-2R expression and IL-2 production by parasite-specific CD8^+^ T cells. This effect seems to involve Ang II-induced NADPH oxidase-mediated reactive oxygen species generation and activation of calcineurin phosphatase (Nataraj et al., 1999; Inoue et al., 2006). In addition to expansion, AT_1_R has also been reported to modulate T-cell activation (Nataraj et al., 1999; Guzik et al., 2007; Jurewicz et al., 2007; Hoch et al., 2008; Platten et al., 2009; Silva-Filho et al., 2011). Here we observed, using both genetic and pharmacological approaches, that AT_1_R induces higher activation and exhaustion of *Plasmodium*-specific CD8^+^ T cells during blood-stage malaria. The higher expression of CD69 and CD44 in AT_1_R-sufficient OT-I cells indicates that AT_1_R confers a higher capacity to respond to antigen, which could contribute to the exhausted phenotype. This also could be correlated with the higher IL-2 production by WT OT-I cells, because CD69 signaling upregulates IL-2 expression via NFAT and AP-1 transcription factors (D'Ambrosio et al., 1993). In turn, CD160 expression increases in cells expressing high levels of CD44 and producing more IFN-γ (Tsujimura et al., 2006). In agreement, CD44 and CD160 are upregulated to a greater degree in AT_1_R-sufficient CD8^+^ T cells, which also produce higher amounts of IFN-γ. -γ. Along with the lower activation, there is a lower percentage of AT_1_R-deficient OT-I cells expressing the exhaustion molecules CTLA-4 and LAG-3. Usually, exhausted T cells lose the ability to produce cytokines such as IL-2, IFN-γ and TNF-α and to degranulate (Wherry and Kurachi, 2015). Here, although AT_1_R^−/−^ OT-I cells are less exhausted, they produce lower amounts of cytokines under re-stimulation. This difference could be because AT_1_R^−/−^ OT-I cells are also less activated.

We also demonstrated the importance of AT_1_R in CCR5 and CXCR3 upregulation in *Plasmodium*-specific CD8^+^ T cells. This could correlate with the AT_1_R-induced migration and sequestration of T cells into the brain microvasculature during ECM (Silva-Filho et al., 2013; Howland et al., 2015a). In this regard, CCR5 expression and their ligand RANTES/CCL5 are upregulated in the brain of patients with CM (Sarfo et al., 2004). In addition, in CCR5-deficient mice, there is a lower number of CD8^+^ T cells accumulated in the cerebral microvasculature and 80% of mice are resistant to PbA-mediated ECM (Belnoue, 2003). Another chemokine receptor involved in ECM pathogenesis is the CXC chemokine receptor 3 (CXCR3) (Hansen et al., 2007; Campanella et al., 2008; Miu et al., 2008; Van den Steen et al., 2008; Nie et al., 2009). CXCR3 expression is upregulated only in T cells from CM-susceptible but not CM-resistant mice, indicating that CXCR3 expression correlates with disease severity (Van den Steen et al., 2008). In agreement, 70–80% of CXCR3^−/−^ mice are resistant to PbA-mediated CM (Campanella et al., 2008; Miu et al., 2008). Thus, the upregulation of the integrin LFA-1 (CD11a), and the chemokine receptors CCR5 and CXCR3 in AT_1_R-sufficient *Plasmodium*-specific CD8^+^ T cells could be the mechanisms involved in AT_1_R-induced sequestration of CD8^+^ T cells in the inflamed tissues during ECM (Silva-Filho et al., 2013; Howland et al., 2015a). The quality of the T-cell response, verified by the ability to produce multiple cytokines and degranulate when in contact with the target cell, is another important parameter for consideration of the effector response (Sandberg et al., 2001; Lichterfeld, 2004; Seder et al., 2008; Thiers, 2008). During blood-stage malaria, degranulation of Granzyme B, perforin, and exacerbated levels of proinflammatory cytokines such as TNF-α and INF-γ are involved with the damage to the blood–brain barrier (Grau et al., 1991; Boubou et al., 1999; Engwerda et al., 2002; Nitcheu et al., 2003; Potter et al., 2006; Suidan et al., 2008; Claser et al., 2011; Haque et al., 2011; Shaw et al., 2015). Here, we verified that along with the higher activation phenotype, WT OT-I cells produce higher amounts of IL-2, TNF-α, and IFN-γ and degranulate in comparison to AT_1_R^−/−^ OT-1 cells.

The present results are similar to previous studies showing that Ang II, via AT_1_R, increases T-cell activation, differentiation into effector cells, adhesion, and migration, leading to the infiltration of T cells in different organs (Nataraj et al., 1999; Inoue et al., 2006; Guzik et al., 2007; Jurewicz et al., 2007; Crowley et al., 2008; Hoch et al., 2008; Platten et al., 2009; Silva-Filho et al., 2011, 2013; Zhang et al., 2012). However, it is important to highlight the observed contrasts between the current findings and our results using immunization with radiation-attenuated *Plasmodium* sporozoites (Silva-Filho et al., 2016). For instance, CD69, CD160, CD44, LAG3, and CTLA-4 expression on day 7 is unaffected in the absence of AT_1_R following immunization (Silva-Filho et al., 2016), but here we showed that the expression of these proteins is reduced by day 6 following infection. These data suggest that there are biologically important context-specific differences in the intrinsic role of AT_1_R in CD8^+^ T cells. Likewise, HO-1 plays distinct roles at different stages of the *Plasmodium* life cycle. During the liver stage, upregulation of HO-1 leads to an increase in parasite liver load (Epiphanio et al., 2008), whereas in mice injected with PbA-infected red blood cells, the establishment of experimental CM is suppressed (Pamplona et al., 2007). Malaria is a very complex disease, with multiple arms of immune system working together and modulating one another. Thus, intrinsic differences between the two experimental models, such as the absolute expression level of the antigen, the location and mechanisms of antigen presentation, are important factors in determining antigen-specific immune responses that could explain such discrepancies (Bagot et al., 2004; Lin et al., 2014). In addition, divergences may also occur due to: (1) the life-long deletion of AT_1_R in T cells might lead to compensatory changes in other genes and related signals, modulating T-cell populations or other properties of specific T cells; (2) AT_1_R can exhibit dual signaling in which G-protein activation leads to deleterious effects, whereas Gα(q) protein-independent/β-arrestin–dependent pathways promote beneficial effects (Zhang et al., 2012); (3) the AT_1_R can heterodimerize with other angiotensin or bradykinin receptors, affecting downstream signaling pathways. In addition, AT_1_R activates multiple downstream signals important to induce pro-inflammatory transcription factors, T-cell activation, proliferation, chemotaxis and cytokine production (Sinclair et al., 2008; Smith-Garvin et al., 2009; Balakumar and Jagadeesh, 2014). However, the AT_1_R-induced signaling pathways behind these processes are not yet known. Thus, the predominance of a set of signaling pathways induced by AT_1_R could diverge during the T-cell response following different immune contexts. Future studies will investigate how AT_1_R signaling leads to transcription of the T-cell molecules following infection vs. immunization, which may indicate additional levels of complexity in the role of AT_1_R in the interplay between host and pathogen.

Previously, it was suggested that increased levels of Ang II have a beneficial effect against malaria-induced pathology in mouse models. A significant reduction in blood parasitemia was observed in mice infected with PbA treated with a supraphysiological concentration of Ang II (Gallego-Delgado et al., 2015). A moderate reduction in the establishment of CM and decreased incidence of brain hemorrhage followed by a modest increase in survival were also verified (Gallego-Delgado et al., 2015). Apparently, these results differ from the current study and our previous works (Silva-Filho et al., 2013, 2016). However, because Ang II is quickly metabolized forming different biologically active peptides, the amelioration of mice could be attributed to increased levels of plasma Ang-(1–7) (Saraiva et al., 2011; Silva et al., 2016a,b). Recently, the same group verified that pharmacological blockade of the AT_1_R or stimulation of AT_2_R protected mice against CM, reduced cerebral hemorrhages and increased survival (Gallego-Delgado et al., 2016). In contrast, AT_2_R-deficient mice were more susceptible to CM. In agreement with this study and our previous works (Silva-Filho et al., 2013, 2016), these observations confirm that Ang II receptors could influence the outcome of experimental CM; protection can be achieved by blockade of AT_1_R or activation of AT_2_R, whereas the opposite effect is observed by activation of AT_1_R when AT_2_R is deleted. Thus, in response to infection during blood-stage malaria, AT_1_R induces the formation of a larger effector population of *Plasmodium*-specific CD8^+^ T cells with a higher capacity to migrate to inflamed tissues, translated by the higher expression of integrin and chemokine receptors, and higher cytokine production. These observations show that inhibition of AT_1_R signaling restricts *Plasmodium*-specific CD8^+^ T-cell function. In addition, because the level of antigen-specific T-cell recruitment to the brain is governed largely by the magnitude of splenic T-cell priming rather than by secondary differences in brain-localized T-cell migratory cues (Lin et al., 2014; Howland et al., 2015a), these data suggest how inhibition of AT_1_R could increase the resistance of mice against CM, promoting survival, improving cognitive parameters, and reducing cerebral edema (Silva-Filho et al., 2013, 2016; Gallego-Delgado et al., 2016). Together, these data bring new contributions to the mechanisms involved in the pathogenic activity of CD8^+^ T cells during the blood-stage of *Plasmodium* infection and to the functions of the RAS in malaria pathogenesis. Repurposing of Ang II modulators, such as AT_1_R antagonists or ACE inhibitors, as adjunctive treatment for CM is a potential therapeutic possibility (Silva-Filho et al., 2013; Gallego-Delgado et al., 2016; Silva et al., 2016b).

## Author contributions

JS performed all the experiments; JS, CC, and AP conceived the project, and JS and AP wrote the manuscript. All authors read and approved the final version.

## Funding

This work was supported by Conselho Nacional de Desenvolvimento Científico e Tecnológico (www.cnpq.br): 1. 57.3695/2008-3 AS, 2. 57.3767/2008-4 CC, 3. 471771/2013-9 CC, 4. 456997/2014-8 AS, and Fundação Carlos Chagas Filho de Amparo à Pesquisa do Estado do Rio de Janeiro (www.faperj.br): 1. E-26/110.551/2010 CC, 2. 111681/2013 CC, 3. E-26/102.170/2013 AS, 4. E-26/201.197/2014 CC.

### Conflict of interest statement

The authors declare that the research was conducted in the absence of any commercial or financial relationships that could be construed as a potential conflict of interest.

## Abbreviations

Ang II: angiotensin II
ACE: angiotensin-converting enzyme
CM: cerebral malaria
CTLA-4: cytotoxic T-lymphocyte-associated protein 4
IFN-γ: interferon-γ
IL-2: interleukin-2
IL-2R: interleukin-2 receptor
IL7-Rα: interleukin-7 receptor α chain
KLRG-1: killer cell lectin-like receptor G1
LAG-3: lymphocyte-activation gene 3
LFA-1: Lymphocyte function-associated antigen 1
LT-α: lymphotoxin-α
MFI: median fluorescence intensity
PD-1: programmed cell death 1
p.i.: post infection
RAS: renin-angiotensin system
TNF-α: tumor necrosis factor-α
WT: wild-type.
