# Supplementary material for: Targeting Angiotensin II Type-1 Receptor (AT1R) Inhibits the Harmful Phenotype of Plasmodium-Specific CD8+ T Cells during Blood-Stage Malaria
Source: Front Cell Infect Microbiol. 2017 Feb 16;7:42. doi: 10.3389/fcimb.2017.00042 (PMC5311040; doi:10.3389/fcimb.2017.00042)
Supplement: Supplementary file 1 [file Image1.PDF]

Supplementary Material

Targeting Angiotensin II type-1 receptor (AT_1R) inhibits the harmful phenotype of *Plasmodium*-specific $CD8^+$ T cells during blood-stage malaria

Joao Luiz Silva-Filho[1,4], Celso Caruso-Neves[1,2], Ana Acacia Sá Pinheiro[1,3,*]

[1]Laboratório de Bioquímica e Sinalização Celular, Instituto de Biofísica Carlos Chagas Filho, Universidade Federal do Rio de Janeiro, Rio de Janeiro, Brazil

[2]Instituto Nacional de Ciência e Tecnologia em Biologia e Bioimagem, Conselho Nacional de Desenvolvimento Científico e Tecnológico/MCT, Rio de Janeiro, Brazil

[3]Instituto Nacional para Pesquisa Translacional em Saúde e Ambiente na Região Amazônica, Conselho Nacional de Desenvolvimento Científico e Tecnológico/MCT, Rio de Janeiro, Brazil

[4]Current affiliation: Laboratory of Tropical Diseases – Prof. Dr. Luiz Jacintho da Silva, Department of Genetics, Evolution and Bioagents, Institute of Biology (IB), University of Campinas (UNICAMP). Campinas, Brazil.

*Corresponding Author: Ana Acacia Sá Pinheiro; Universidade Federal do Rio de Janeiro, CCS, bloco C, sala 34, 21941–902, Rio de Janeiro, RJ, Brazil. Tel.: +55 21 3938 6582; fax: +55 21 2280 8193. E-mail: acacia@biof.ufrj.br

1. Supplementary Figures

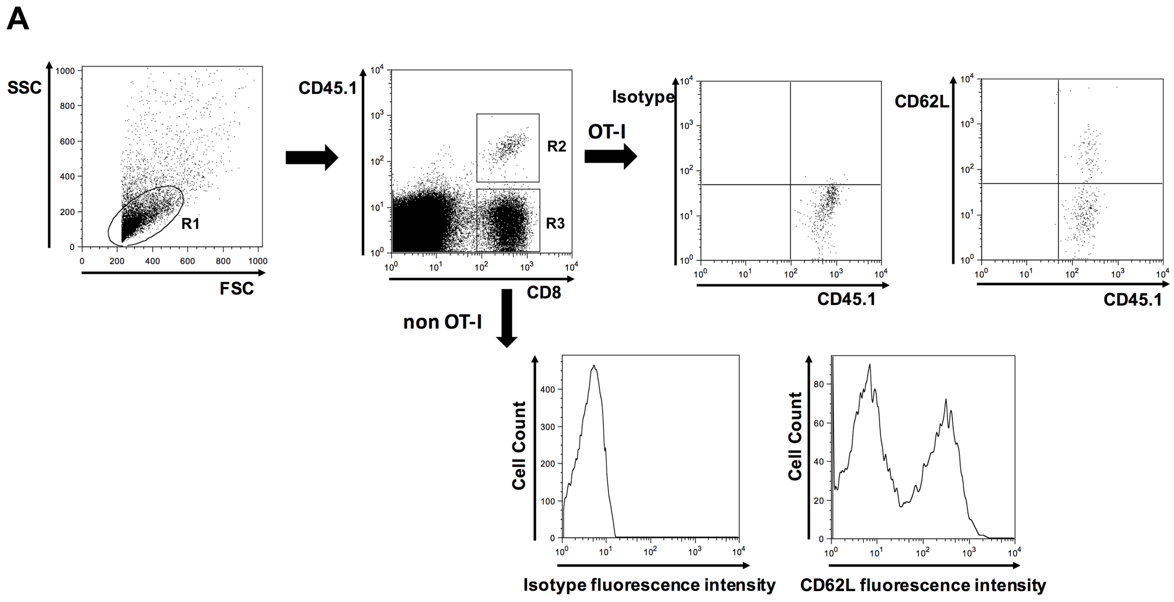

Figure S1. Schematics of OT-I (CD8$^+$CD45.1$^+$Vα2$^+$ cells) gating strategy. (A) Gating strategy used for flow cytometry analysis. A lymphocyte gate (R1) was defined based on the cells' forward scatter (FSC) and side scatter (SSC). Further, gated on CD8$^+$CD45.1$^+$ lymphocytes (R2, OT-I cells) or CD8$^+$CD45.1$^-$ lymphocytes (R3, endogenous non-OT-I cells) and analyzed for the percentage and levels of expression (based on the MFI) of different proteins (e.g., CD62L), using corresponding isotypes as irrelevant antibodies to define positive populations.
