# Supplementary material for: Targeting Angiotensin II Type-1 Receptor (AT1R) Inhibits the Harmful Phenotype of Plasmodium-Specific CD8+ T Cells during Blood-Stage Malaria
Source: Front Cell Infect Microbiol. 2017 Feb 16;7:42. doi: 10.3389/fcimb.2017.00042 (PMC5311040; doi:10.3389/fcimb.2017.00042)
Supplement: Supplementary file 2 [file Image2.PDF]

*Supplementary Material*

# Targeting Angiotensin II type-1 receptor ($AT_1R$) inhibits the harmful phenotype of *Plasmodium*-specific $CD8^+$ T cells during blood-stage malaria

Joao Luiz Silva-Filho[1,4], Celso Caruso-Neves[1,2], Ana Acacia Sá Pinheiro[1,3,*]

[1]Laboratório de Bioquímica e Sinalização Celular, Instituto de Biofísica Carlos Chagas Filho, Universidade Federal do Rio de Janeiro, Rio de Janeiro, Brazil

[2]Instituto Nacional de Ciência e Tecnologia em Biologia e Bioimagem, Conselho Nacional de Desenvolvimento Científico e Tecnológico/MCT, Rio de Janeiro, Brazil

[3]Instituto Nacional para Pesquisa Translacional em Saúde e Ambiente na Região Amazônica, Conselho Nacional de Desenvolvimento Científico e Tecnológico/MCT, Rio de Janeiro, Brazil

[4]Current affiliation: Laboratory of Tropical Diseases – Prof. Dr. Luiz Jacintho da Silva, Department of Genetics, Evolution and Bioagents, Institute of Biology (IB), University of Campinas (UNICAMP). Campinas, Brazil.

*Corresponding Author: Ana Acacia Sá Pinheiro; Universidade Federal do Rio de Janeiro, CCS, bloco C, sala 34, 21941–902, Rio de Janeiro, RJ, Brazil. Tel.: +55 21 3938 6582; fax: +55 21 2280 8193. E-mail: acacia@biof.ufrj.br

1. Supplementary Figures

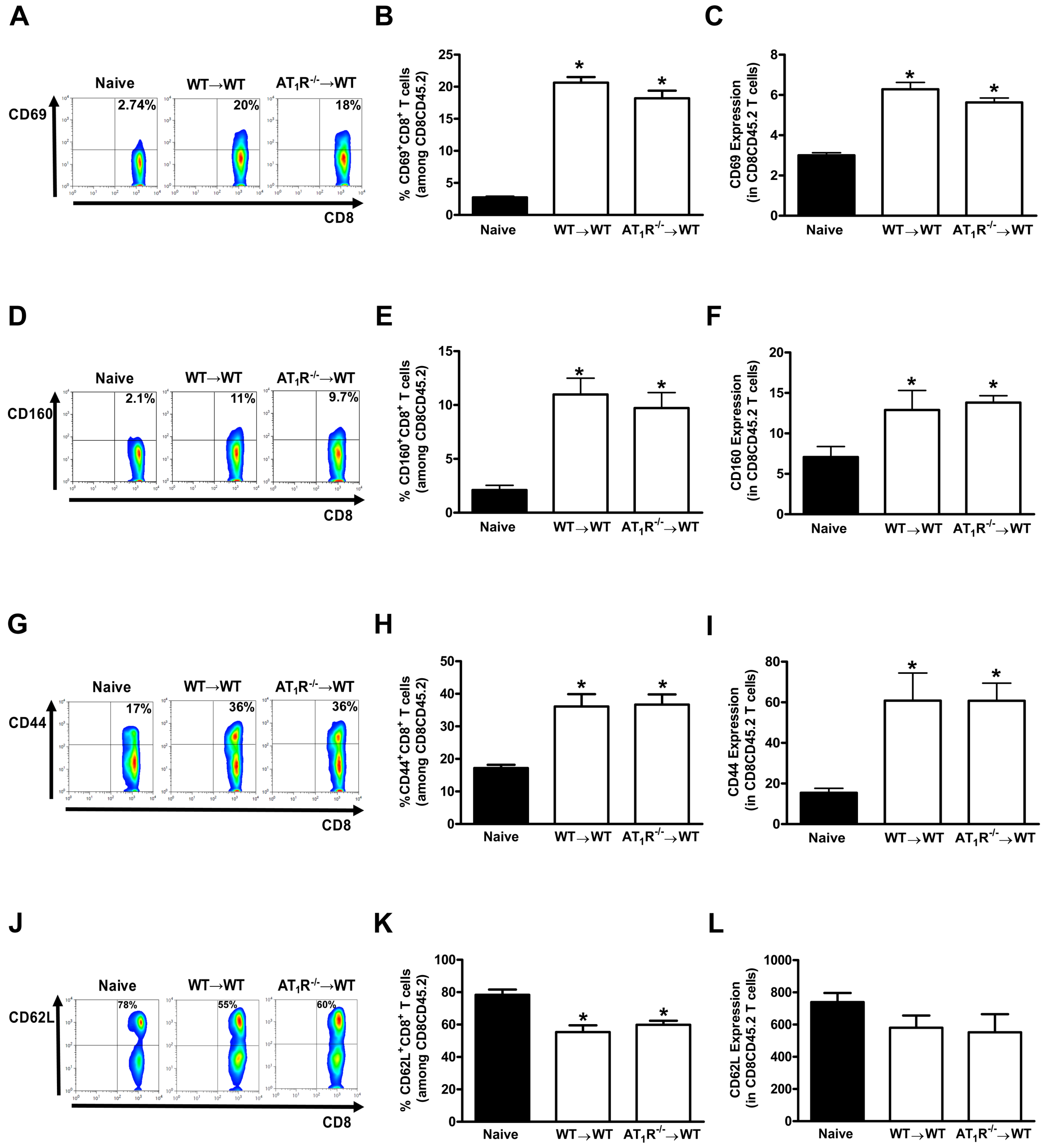


**Figure S2.** Low level of activation of endogenous polyclonal CD8$^+$ T cells in infected mice containing WT or AT$_1$R-deficient OT-I cells. Percentages and expression of the markers of activation (A–C) CD69, (D–F) CD160, (G–I) CD160, and (J–L) CD62L were evaluated in the endogenous polyclonal CD8$^+$ T cells (non-OT-I cells; CD8$^+$CD45.1$^-$) at day 6 post infection. The cells were recovered from the spleen of naive mice and mice infected with OVA-PbA that received WT (WT→WT) or

$AT_1R^{-/-}$ OT-I cells ($AT_1R^{-/-}$→WT). The gating strategy used for the flow cytometry analysis is indicated in the Materials and methods section. Data are means ± SEM of five mice per group and are representative of three independent experiments with similar results for each indicated time point. *Statistically significant compared with values for naive mice ($p<0.05$)
